# Supplementary figures and images for: The two types of society: Computationally revealing recurrent social formations and their evolutionary trajectories
Source: PLoS One. 2020 May 13;15(5):e0232609. doi: 10.1371/journal.pone.0232609 (PMC7219743; doi:10.1371/journal.pone.0232609)

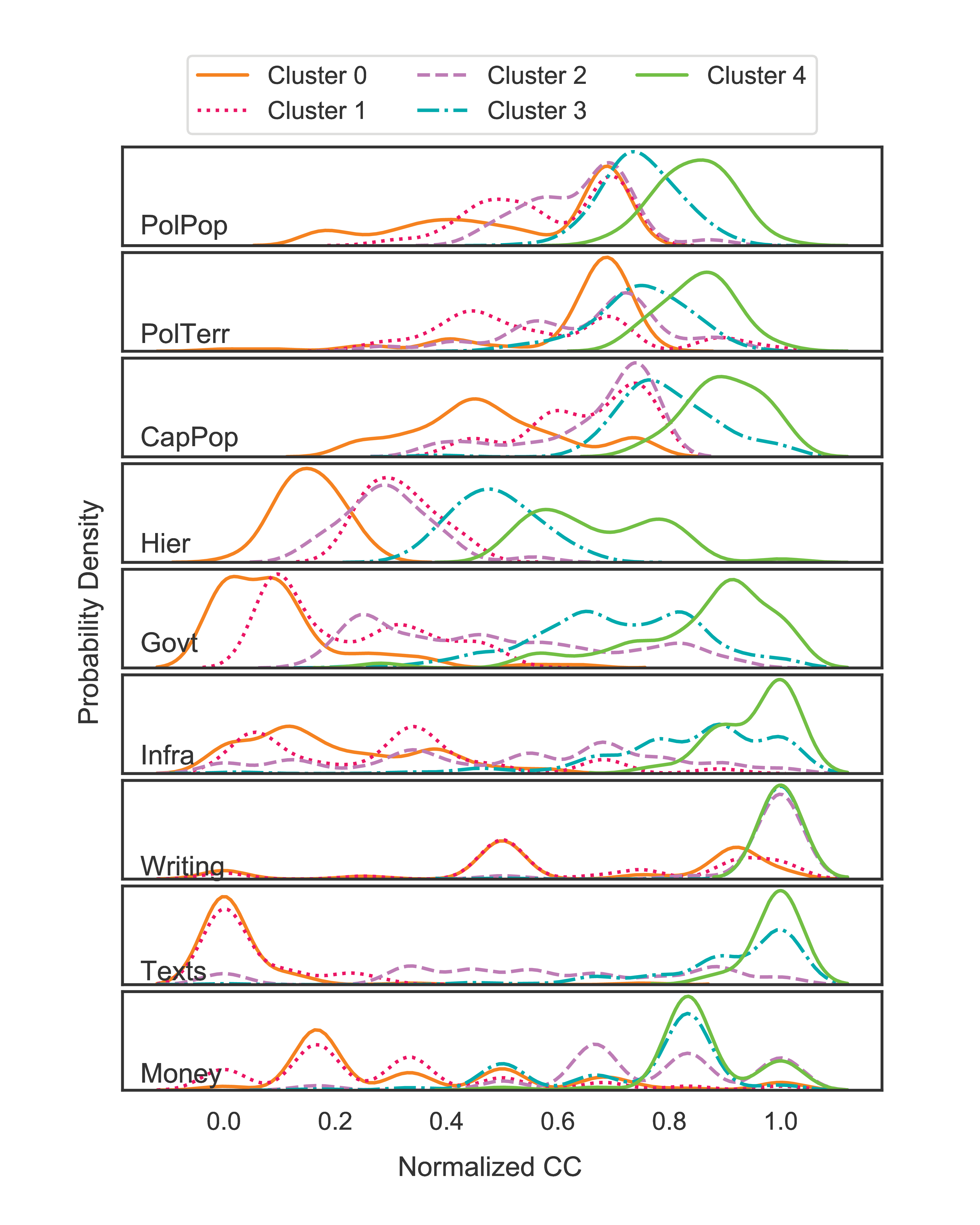

Supplement: S1 Fig — Using a kernel-density estimation. (TIF) [file pone.0232609.s001.tif]

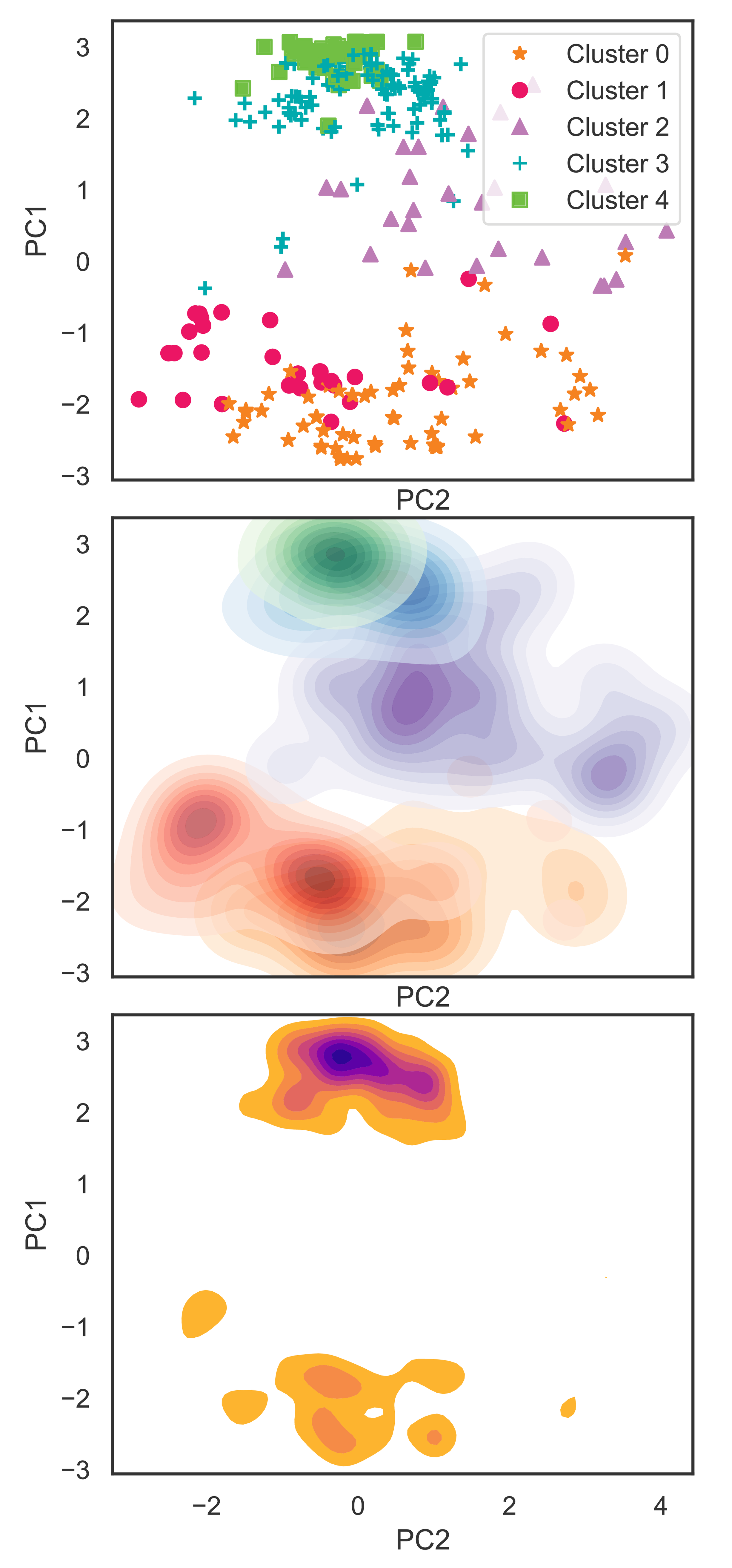

Supplement: S2 Fig — (TIF) [file pone.0232609.s002.tif]

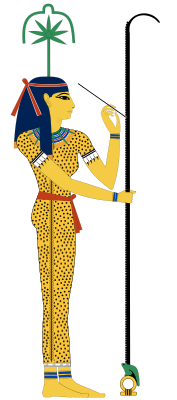

Supplement: S2 File — We include our Python 3 program that begins with the original, untampered Seshat database and performs the entire process of turning it into Shiny Seshat (including all error correction, Complexity Characteristic creation, imputation of missing values, etc.). (ZIP) [file pone.0232609.s004.zip › docs/seshat.png]

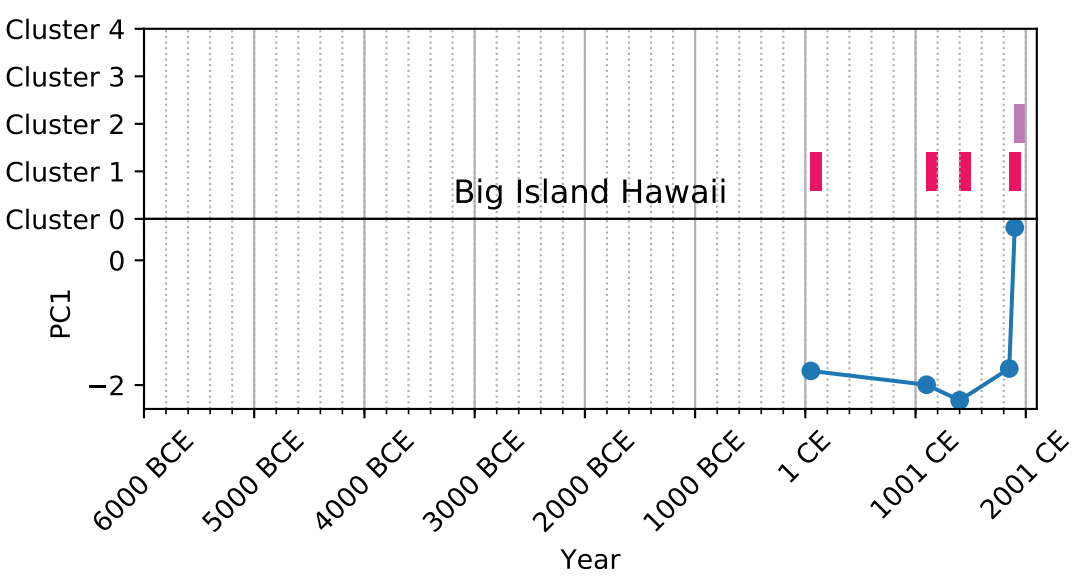

Supplement: S3 File — We include trajectories for all Natural Geographic Areas (NGAs) for which there is sufficient data (all polities with at least 75% complete encoding for the 51 features of analysis; see the Data and methods section for details). (ZIP) [file pone.0232609.s005.zip › traj_Big_Island_Hawaii.pdf]

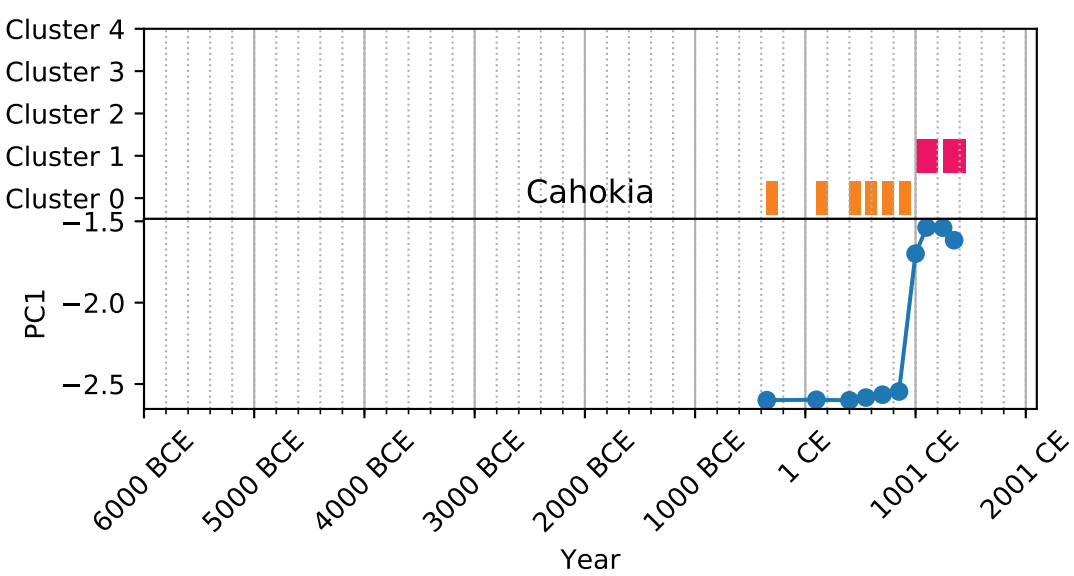

Supplement: S3 File — We include trajectories for all Natural Geographic Areas (NGAs) for which there is sufficient data (all polities with at least 75% complete encoding for the 51 features of analysis; see the Data and methods section for details). (ZIP) [file pone.0232609.s005.zip › traj_Cahokia.pdf]

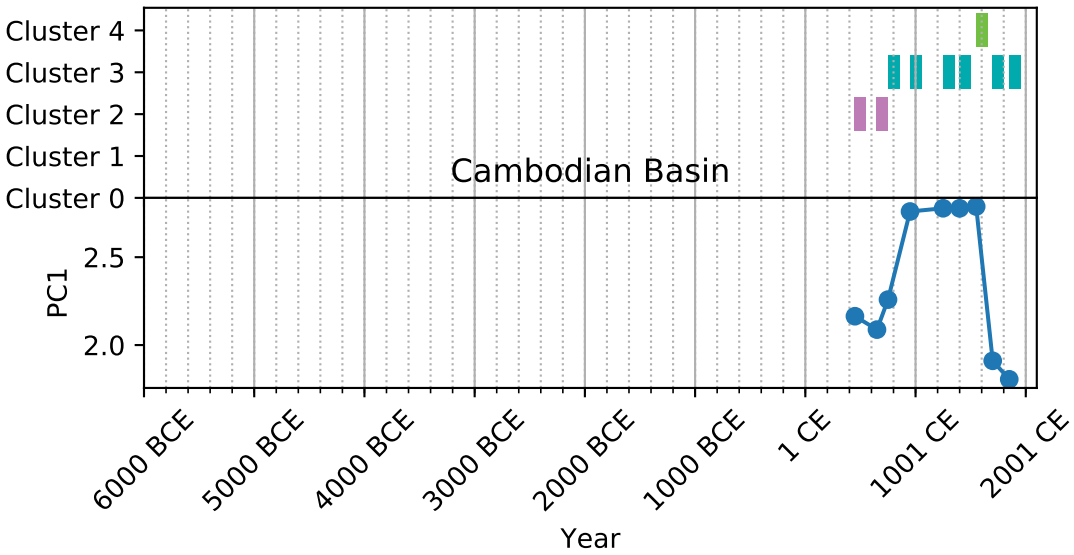

Supplement: S3 File — We include trajectories for all Natural Geographic Areas (NGAs) for which there is sufficient data (all polities with at least 75% complete encoding for the 51 features of analysis; see the Data and methods section for details). (ZIP) [file pone.0232609.s005.zip › traj_Cambodian_Basin.pdf]

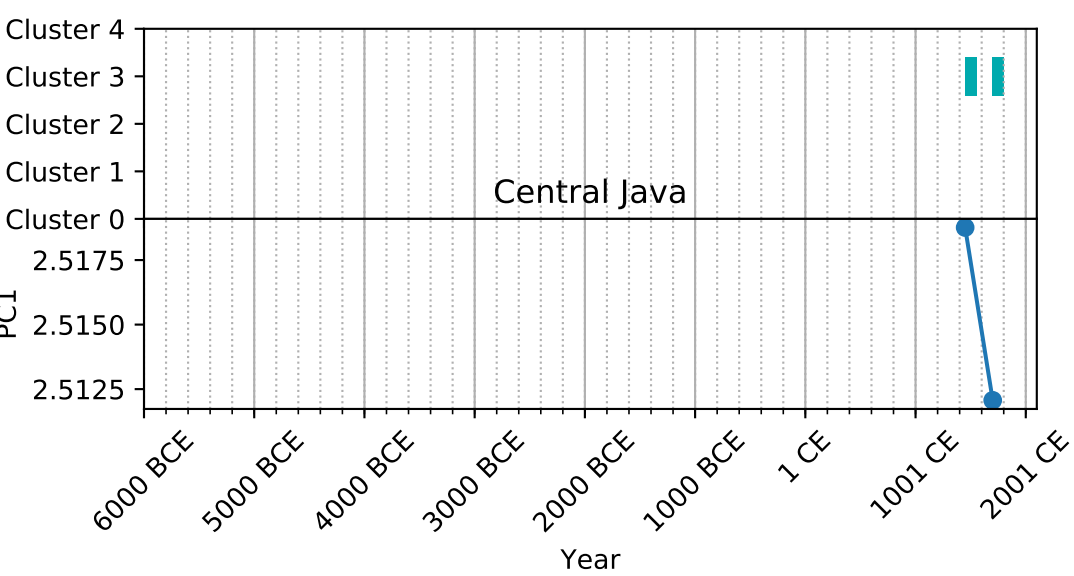

Supplement: S3 File — We include trajectories for all Natural Geographic Areas (NGAs) for which there is sufficient data (all polities with at least 75% complete encoding for the 51 features of analysis; see the Data and methods section for details). (ZIP) [file pone.0232609.s005.zip › traj_Central_Java.pdf]

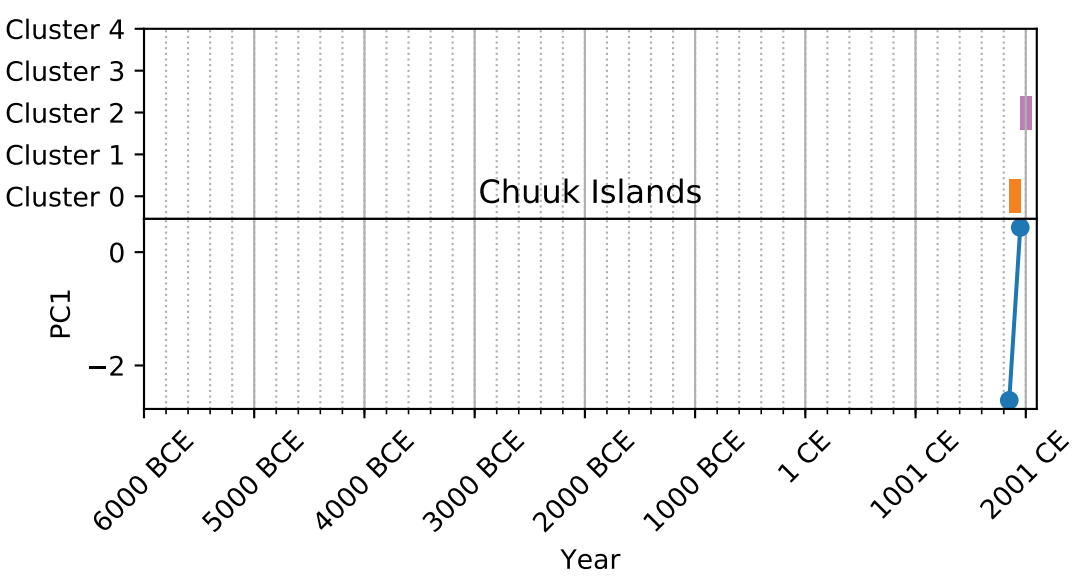

Supplement: S3 File — We include trajectories for all Natural Geographic Areas (NGAs) for which there is sufficient data (all polities with at least 75% complete encoding for the 51 features of analysis; see the Data and methods section for details). (ZIP) [file pone.0232609.s005.zip › traj_Chuuk_Islands.pdf]

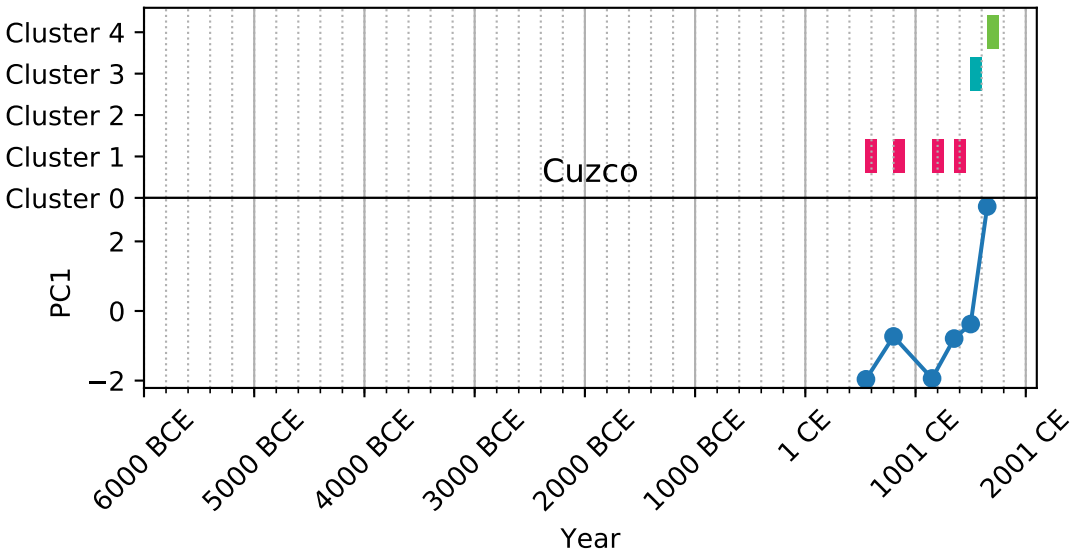

Supplement: S3 File — We include trajectories for all Natural Geographic Areas (NGAs) for which there is sufficient data (all polities with at least 75% complete encoding for the 51 features of analysis; see the Data and methods section for details). (ZIP) [file pone.0232609.s005.zip › traj_Cuzco.pdf]

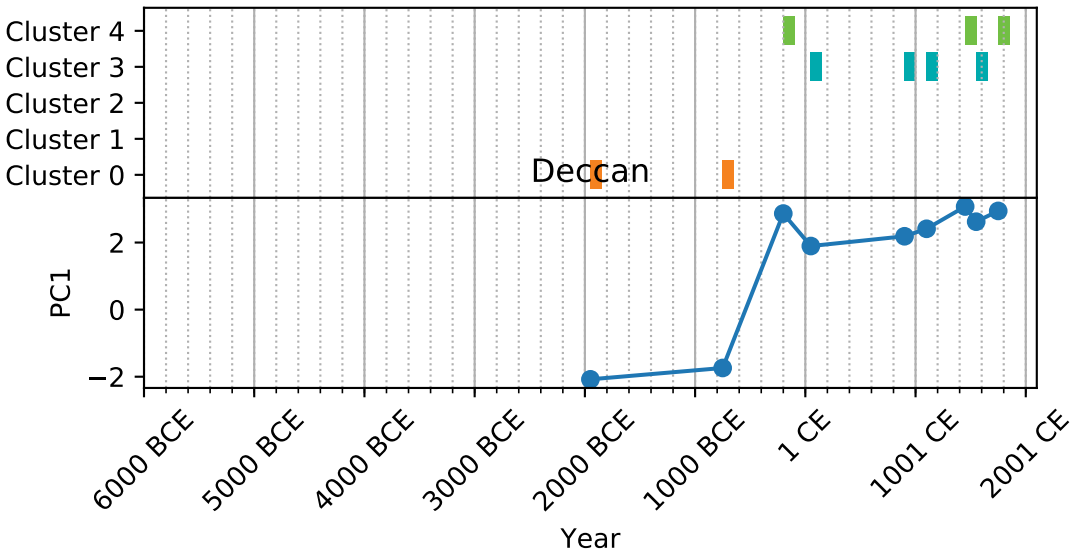

Supplement: S3 File — We include trajectories for all Natural Geographic Areas (NGAs) for which there is sufficient data (all polities with at least 75% complete encoding for the 51 features of analysis; see the Data and methods section for details). (ZIP) [file pone.0232609.s005.zip › traj_Deccan.pdf]

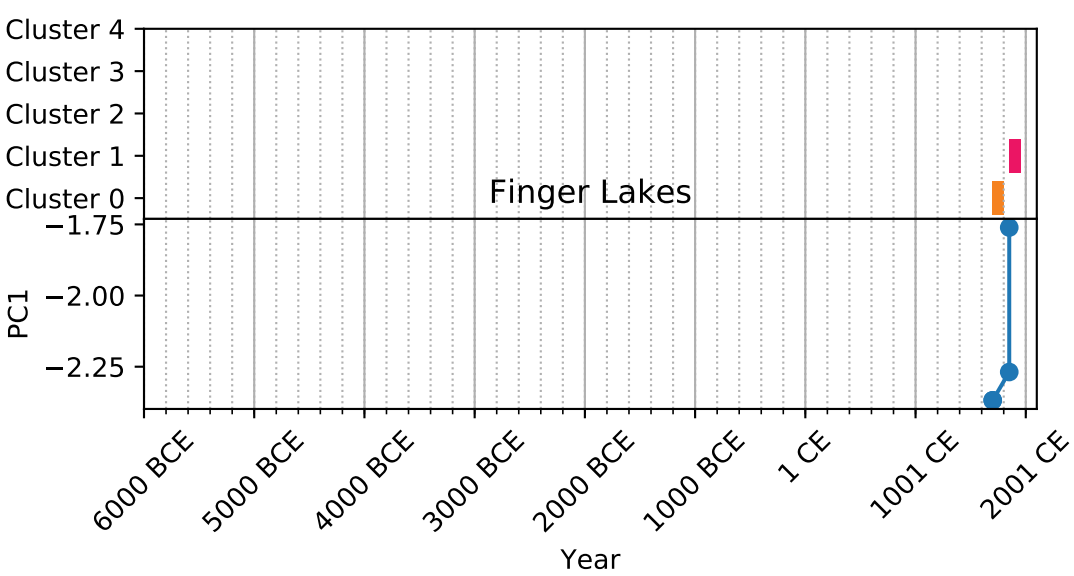

Supplement: S3 File — We include trajectories for all Natural Geographic Areas (NGAs) for which there is sufficient data (all polities with at least 75% complete encoding for the 51 features of analysis; see the Data and methods section for details). (ZIP) [file pone.0232609.s005.zip › traj_Finger_Lakes.pdf]

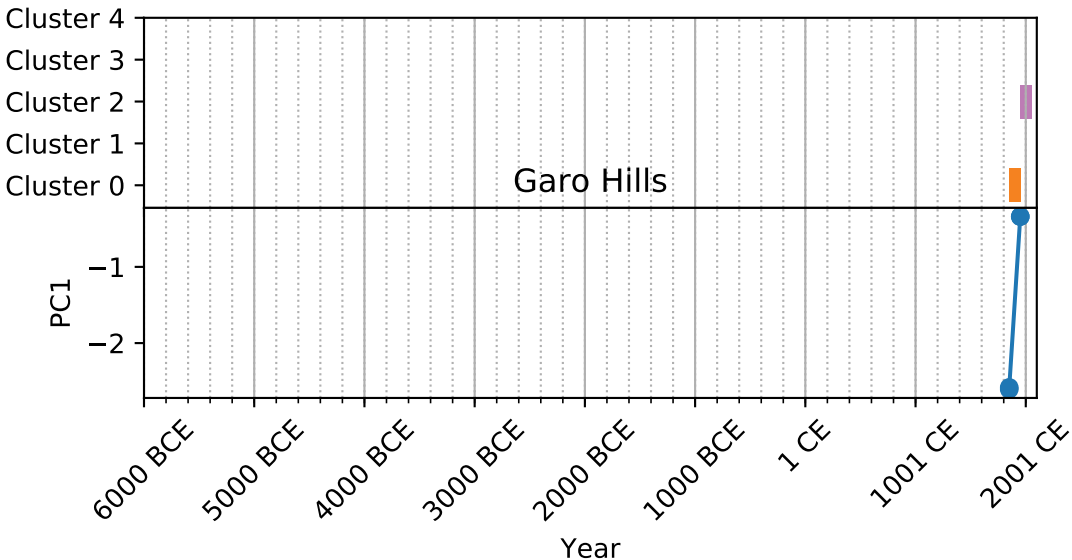

Supplement: S3 File — We include trajectories for all Natural Geographic Areas (NGAs) for which there is sufficient data (all polities with at least 75% complete encoding for the 51 features of analysis; see the Data and methods section for details). (ZIP) [file pone.0232609.s005.zip › traj_Garo_Hills.pdf]

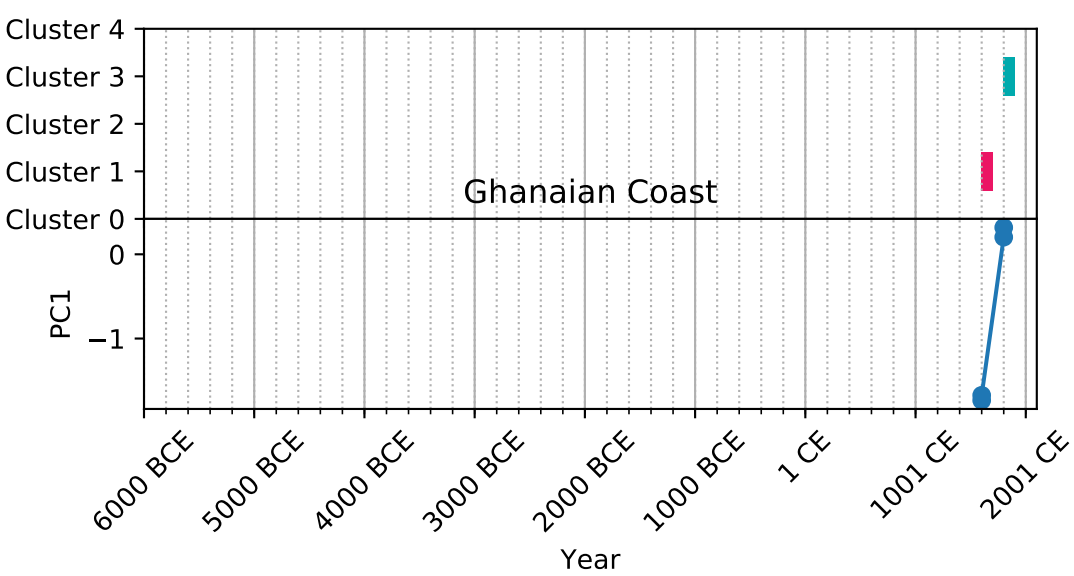

Supplement: S3 File — We include trajectories for all Natural Geographic Areas (NGAs) for which there is sufficient data (all polities with at least 75% complete encoding for the 51 features of analysis; see the Data and methods section for details). (ZIP) [file pone.0232609.s005.zip › traj_Ghanaian_Coast.pdf]

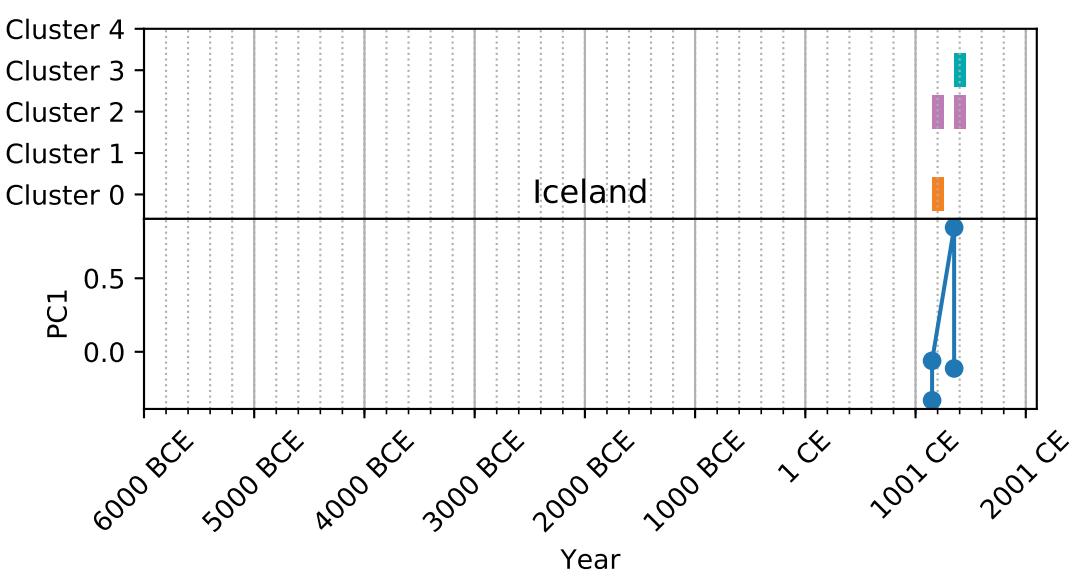

Supplement: S3 File — We include trajectories for all Natural Geographic Areas (NGAs) for which there is sufficient data (all polities with at least 75% complete encoding for the 51 features of analysis; see the Data and methods section for details). (ZIP) [file pone.0232609.s005.zip › traj_Iceland.pdf]

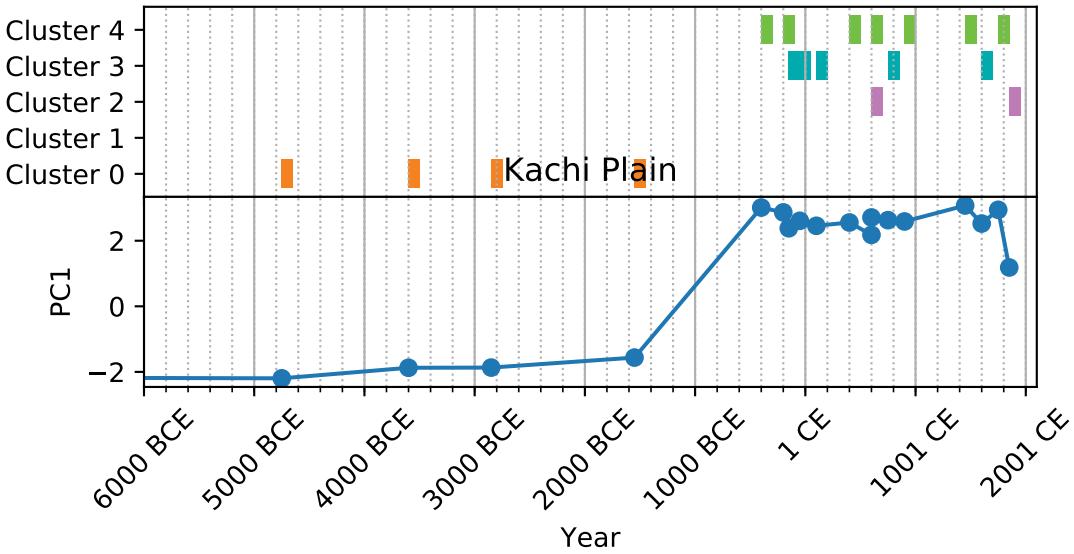

Supplement: S3 File — We include trajectories for all Natural Geographic Areas (NGAs) for which there is sufficient data (all polities with at least 75% complete encoding for the 51 features of analysis; see the Data and methods section for details). (ZIP) [file pone.0232609.s005.zip › traj_Kachi_Plain.pdf]

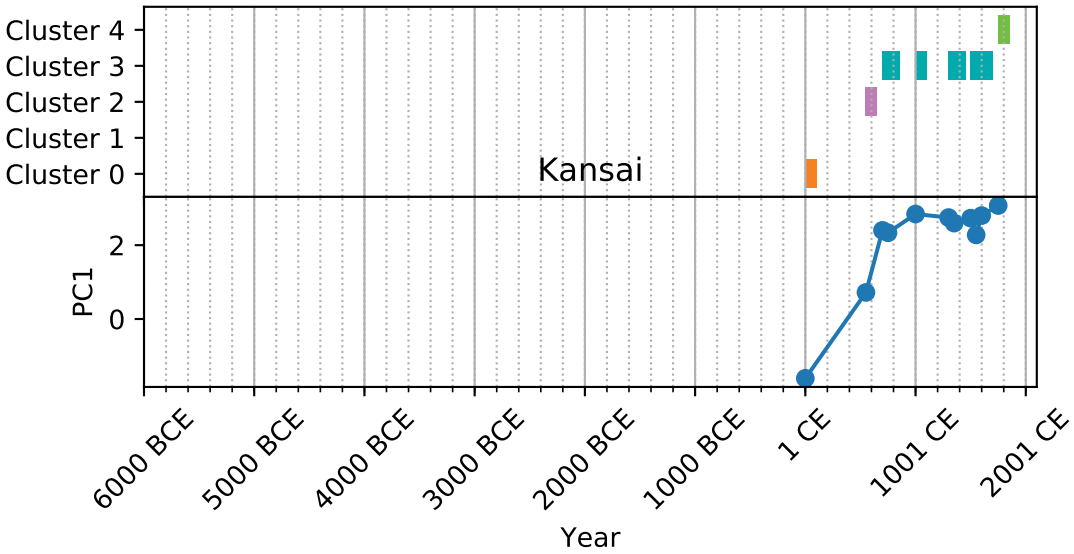

Supplement: S3 File — We include trajectories for all Natural Geographic Areas (NGAs) for which there is sufficient data (all polities with at least 75% complete encoding for the 51 features of analysis; see the Data and methods section for details). (ZIP) [file pone.0232609.s005.zip › traj_Kansai.pdf]

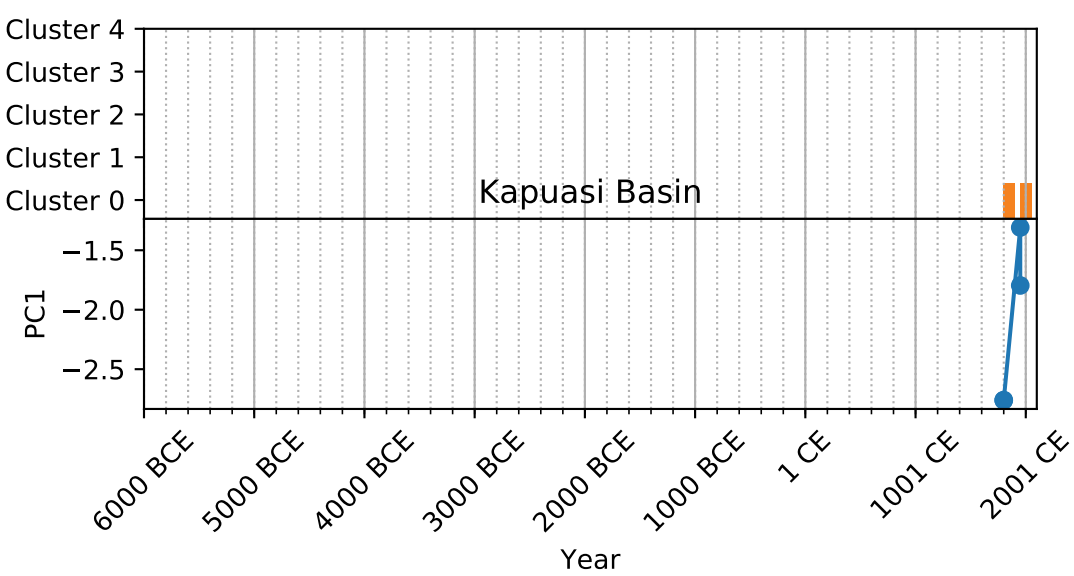

Supplement: S3 File — We include trajectories for all Natural Geographic Areas (NGAs) for which there is sufficient data (all polities with at least 75% complete encoding for the 51 features of analysis; see the Data and methods section for details). (ZIP) [file pone.0232609.s005.zip › traj_Kapuasi_Basin.pdf]

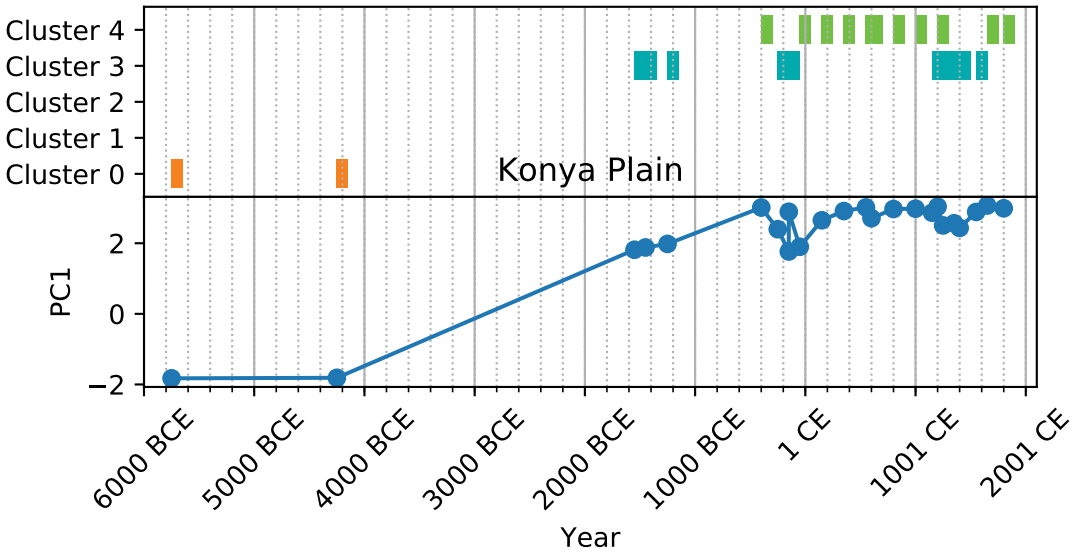

Supplement: S3 File — We include trajectories for all Natural Geographic Areas (NGAs) for which there is sufficient data (all polities with at least 75% complete encoding for the 51 features of analysis; see the Data and methods section for details). (ZIP) [file pone.0232609.s005.zip › traj_Konya_Plain.pdf]

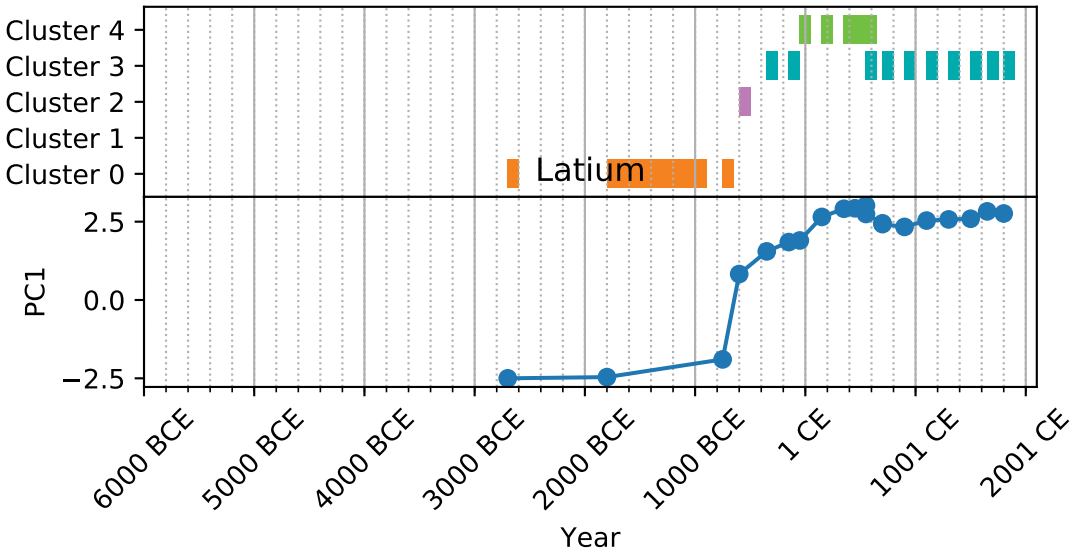

Supplement: S3 File — We include trajectories for all Natural Geographic Areas (NGAs) for which there is sufficient data (all polities with at least 75% complete encoding for the 51 features of analysis; see the Data and methods section for details). (ZIP) [file pone.0232609.s005.zip › traj_Latium.pdf]

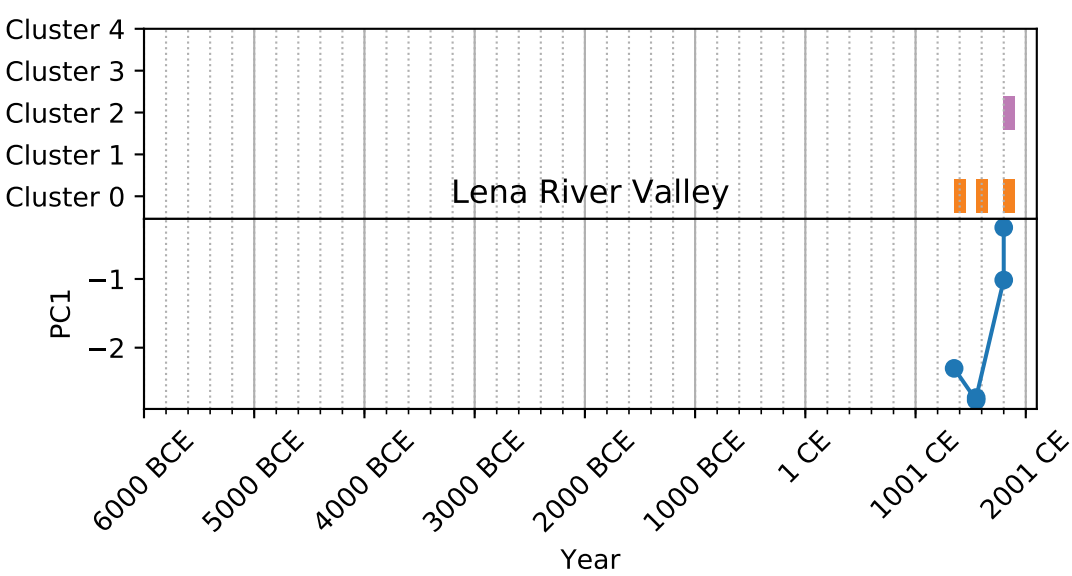

Supplement: S3 File — We include trajectories for all Natural Geographic Areas (NGAs) for which there is sufficient data (all polities with at least 75% complete encoding for the 51 features of analysis; see the Data and methods section for details). (ZIP) [file pone.0232609.s005.zip › traj_Lena_River_Valley.pdf]

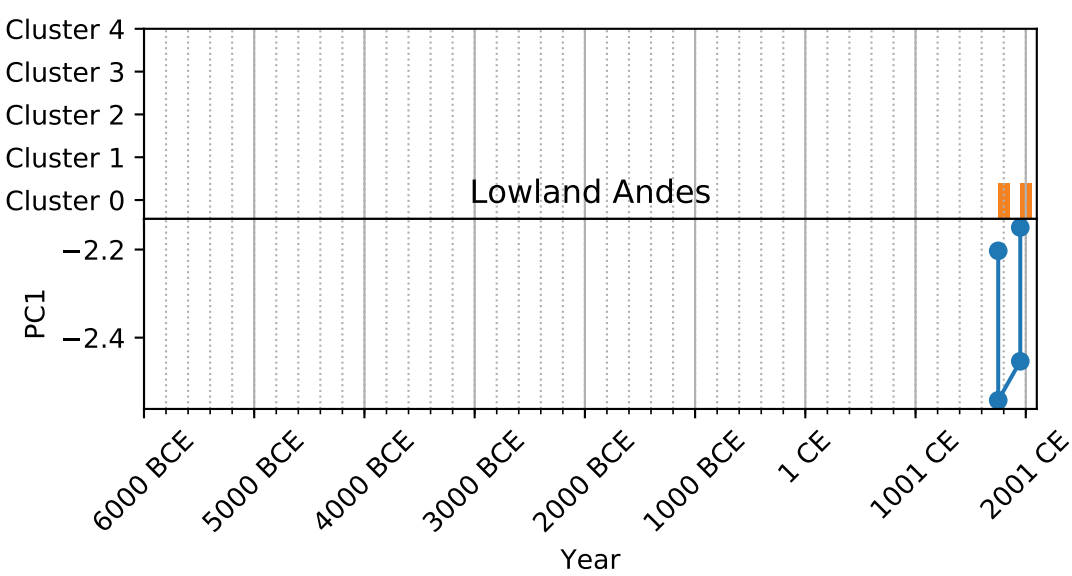

Supplement: S3 File — We include trajectories for all Natural Geographic Areas (NGAs) for which there is sufficient data (all polities with at least 75% complete encoding for the 51 features of analysis; see the Data and methods section for details). (ZIP) [file pone.0232609.s005.zip › traj_Lowland_Andes.pdf]

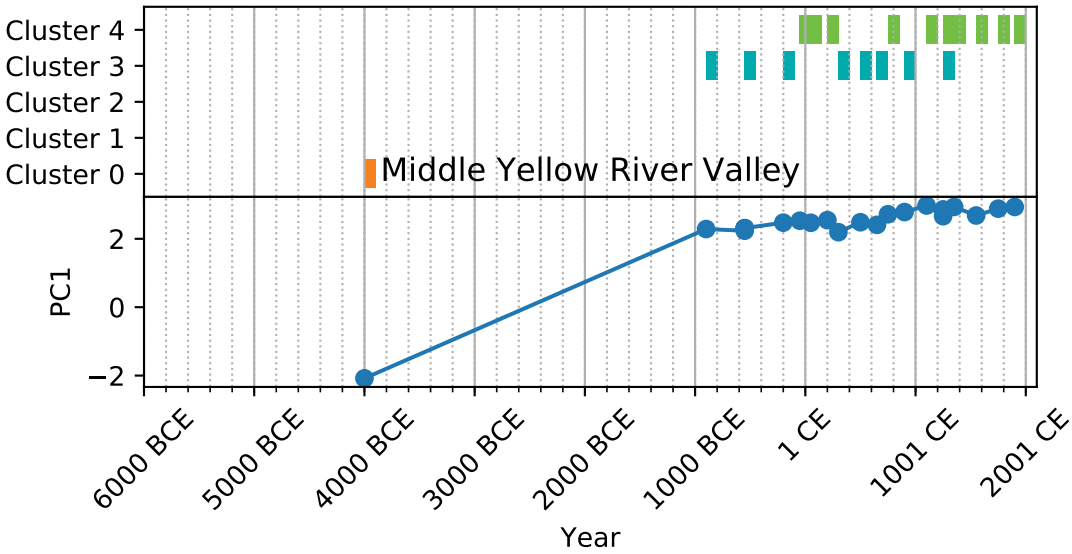

Supplement: S3 File — We include trajectories for all Natural Geographic Areas (NGAs) for which there is sufficient data (all polities with at least 75% complete encoding for the 51 features of analysis; see the Data and methods section for details). (ZIP) [file pone.0232609.s005.zip › traj_Middle_Yellow_River_Valley.pdf]

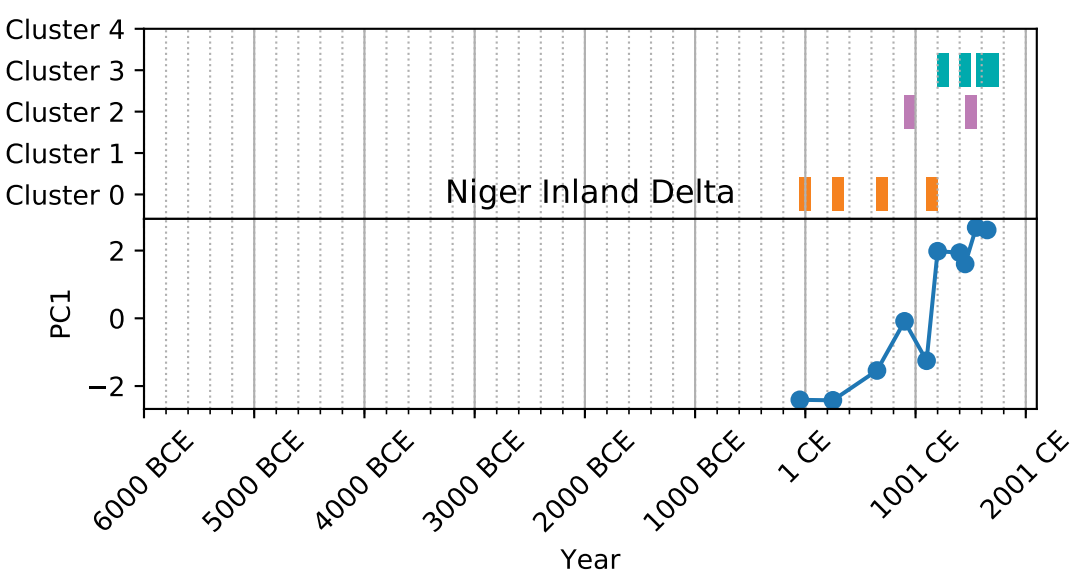

Supplement: S3 File — We include trajectories for all Natural Geographic Areas (NGAs) for which there is sufficient data (all polities with at least 75% complete encoding for the 51 features of analysis; see the Data and methods section for details). (ZIP) [file pone.0232609.s005.zip › traj_Niger_Inland_Delta.pdf]

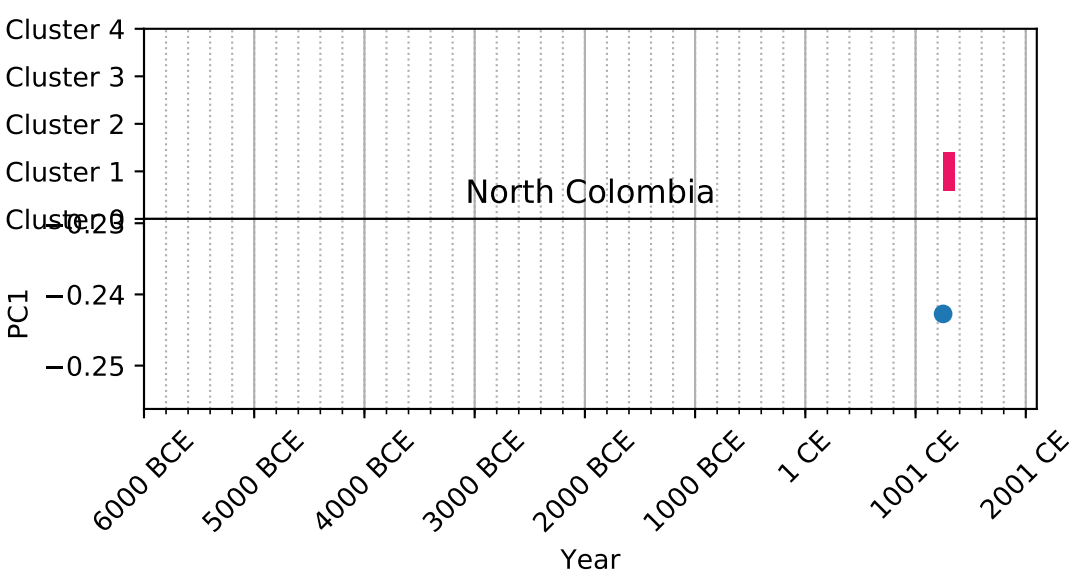

Supplement: S3 File — We include trajectories for all Natural Geographic Areas (NGAs) for which there is sufficient data (all polities with at least 75% complete encoding for the 51 features of analysis; see the Data and methods section for details). (ZIP) [file pone.0232609.s005.zip › traj_North_Colombia.pdf]

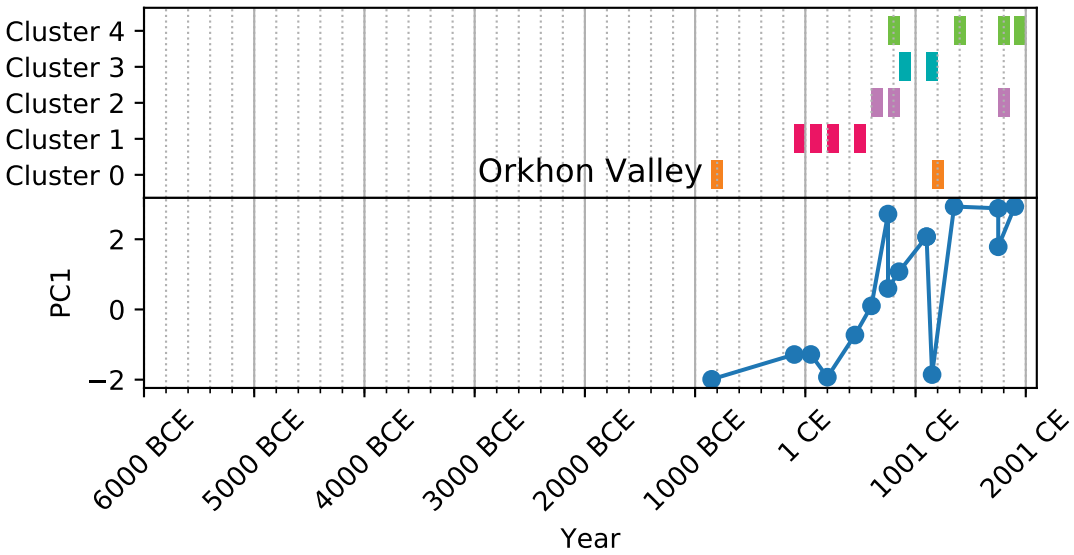

Supplement: S3 File — We include trajectories for all Natural Geographic Areas (NGAs) for which there is sufficient data (all polities with at least 75% complete encoding for the 51 features of analysis; see the Data and methods section for details). (ZIP) [file pone.0232609.s005.zip › traj_Orkhon_Valley.pdf]

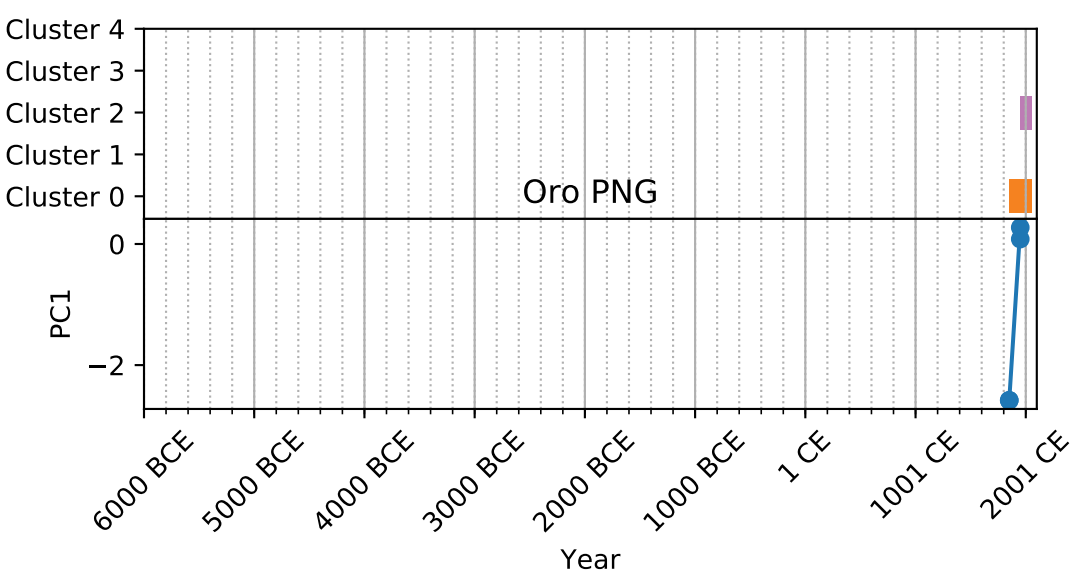

Supplement: S3 File — We include trajectories for all Natural Geographic Areas (NGAs) for which there is sufficient data (all polities with at least 75% complete encoding for the 51 features of analysis; see the Data and methods section for details). (ZIP) [file pone.0232609.s005.zip › traj_Oro_PNG.pdf]

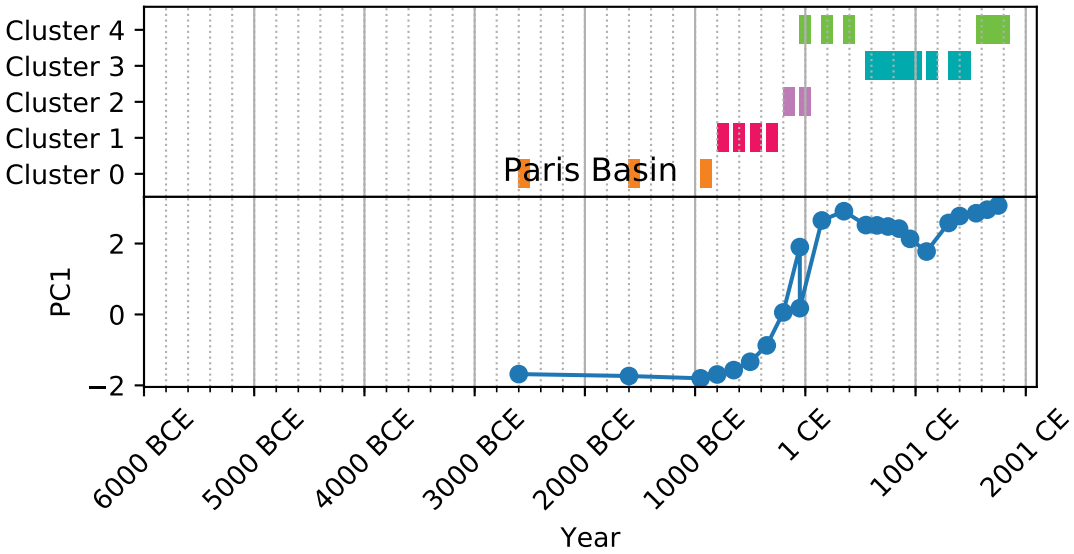

Supplement: S3 File — We include trajectories for all Natural Geographic Areas (NGAs) for which there is sufficient data (all polities with at least 75% complete encoding for the 51 features of analysis; see the Data and methods section for details). (ZIP) [file pone.0232609.s005.zip › traj_Paris_Basin.pdf]

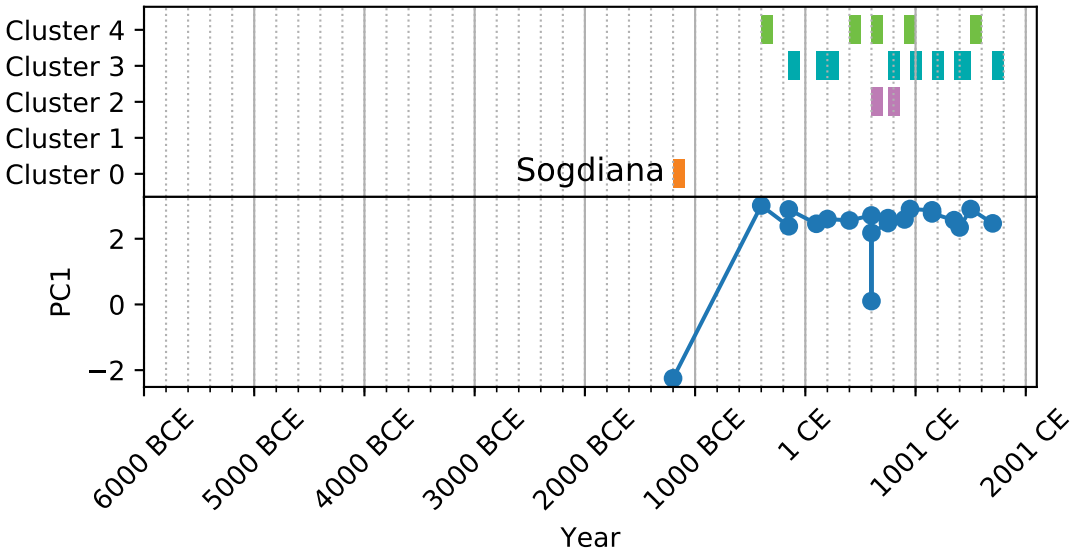

Supplement: S3 File — We include trajectories for all Natural Geographic Areas (NGAs) for which there is sufficient data (all polities with at least 75% complete encoding for the 51 features of analysis; see the Data and methods section for details). (ZIP) [file pone.0232609.s005.zip › traj_Sogdiana.pdf]

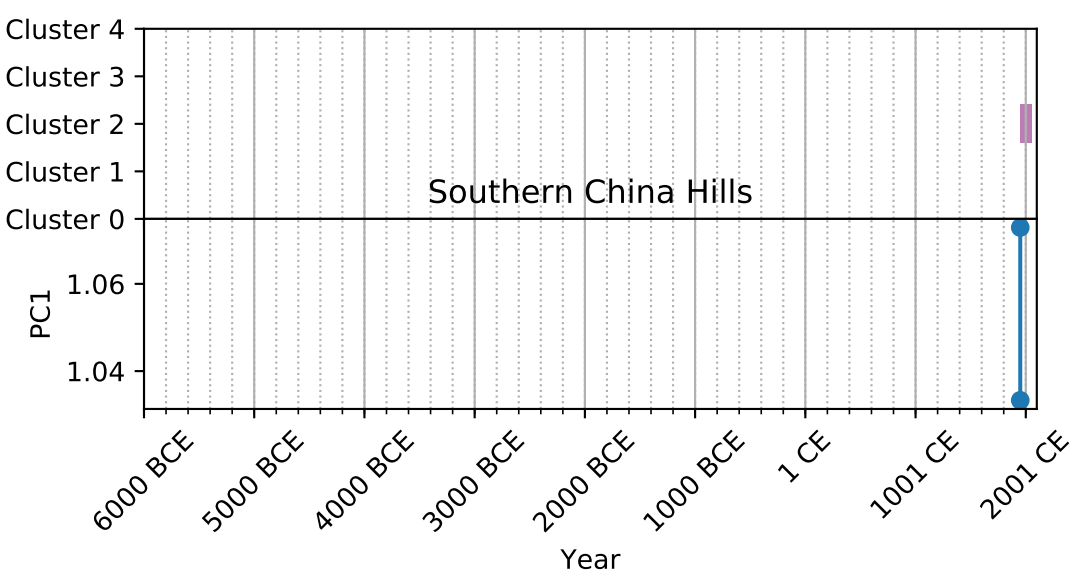

Supplement: S3 File — We include trajectories for all Natural Geographic Areas (NGAs) for which there is sufficient data (all polities with at least 75% complete encoding for the 51 features of analysis; see the Data and methods section for details). (ZIP) [file pone.0232609.s005.zip › traj_Southern_China_Hills.pdf]

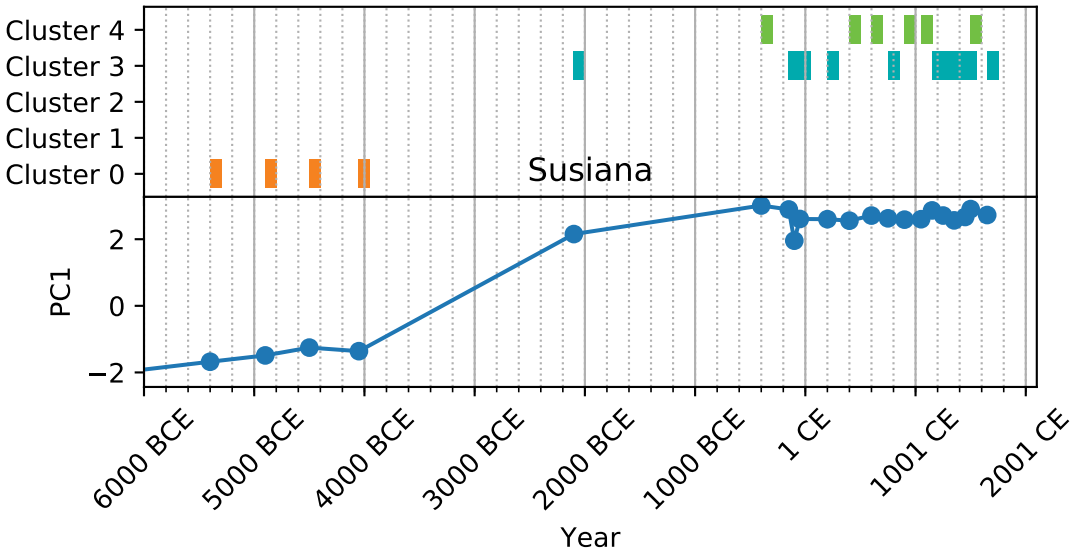

Supplement: S3 File — We include trajectories for all Natural Geographic Areas (NGAs) for which there is sufficient data (all polities with at least 75% complete encoding for the 51 features of analysis; see the Data and methods section for details). (ZIP) [file pone.0232609.s005.zip › traj_Susiana.pdf]

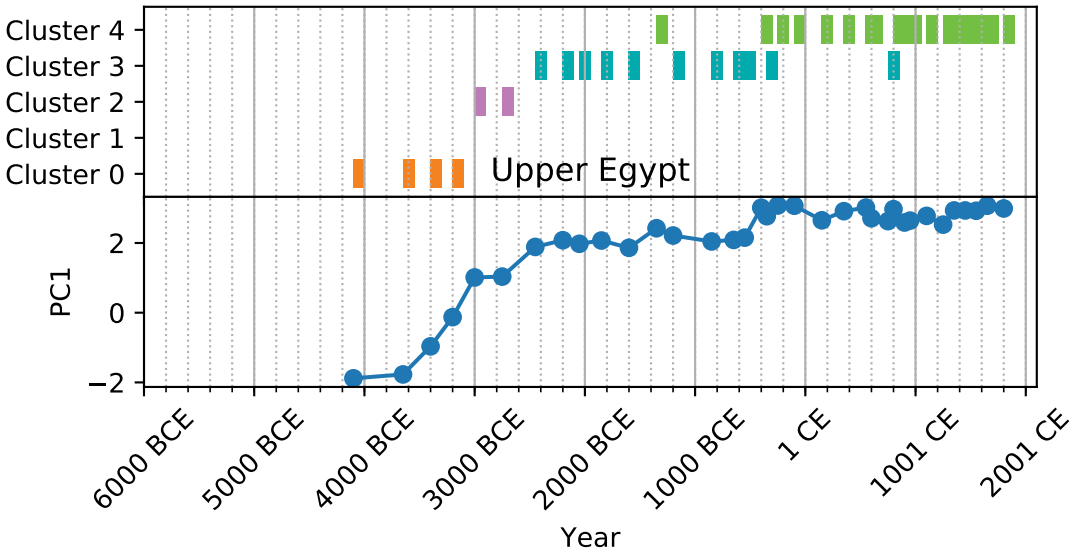

Supplement: S3 File — We include trajectories for all Natural Geographic Areas (NGAs) for which there is sufficient data (all polities with at least 75% complete encoding for the 51 features of analysis; see the Data and methods section for details). (ZIP) [file pone.0232609.s005.zip › traj_Upper_Egypt.pdf]

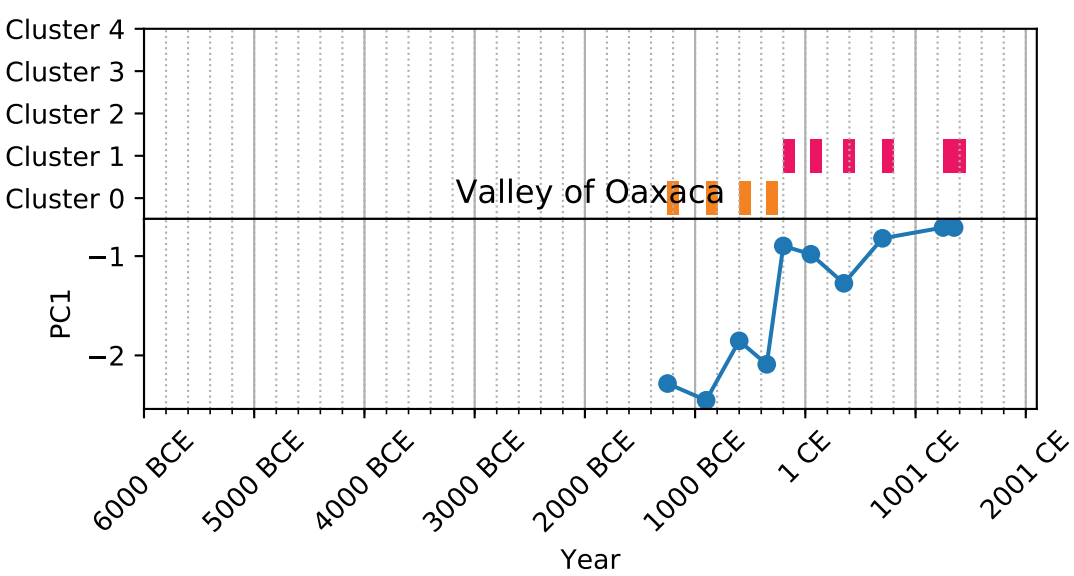

Supplement: S3 File — We include trajectories for all Natural Geographic Areas (NGAs) for which there is sufficient data (all polities with at least 75% complete encoding for the 51 features of analysis; see the Data and methods section for details). (ZIP) [file pone.0232609.s005.zip › traj_Valley_of_Oaxaca.pdf]

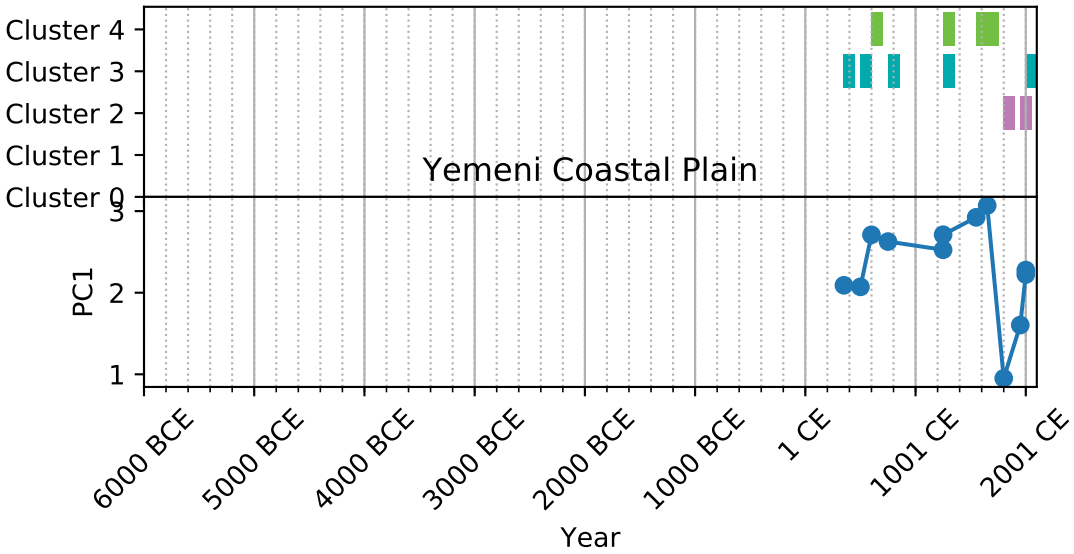

Supplement: S3 File — We include trajectories for all Natural Geographic Areas (NGAs) for which there is sufficient data (all polities with at least 75% complete encoding for the 51 features of analysis; see the Data and methods section for details). (ZIP) [file pone.0232609.s005.zip › traj_Yemeni_Coastal_Plain.pdf]
