# Supplementary material for: The two types of society: Computationally revealing recurrent social formations and their evolutionary trajectories
Source: PLoS One. 2020 May 13;15(5):e0232609. doi: 10.1371/journal.pone.0232609 (PMC7219743; doi:10.1371/journal.pone.0232609)
Supplement: S1 Appendix — (PDF) [file pone.0232609.s006.pdf]

The two types of society: computationally revealing recurrent social formations and their evolutionary trajectories

Lux Miranda, Jacob Freeman

Anthropology Program, Utah State University, Logan, Utah, USA

Appendix: Principal components

In the original principal component analysis (PCA) of Seshat conducted by Turchin and colleagues [1], the first principal component (PC1) was found to be the only significant PC as it was the only one with an eigenvalue above the standard threshold of 1.0. Our replicated PCA using Shiny Seshat, however, provides us with a significant PC1 but also a PC2 eigenvalue of 1.42 — just above the significance threshold (Table 1).

The loadings across PC1 appears to also be less consistent than the prior analysis (Table 2). This may be an artifact of differences in imputation procedure. Loadings for PC2 indicate that it is primarily encoding the *Money* complexity characteristic (CC) and secondarily encoding the *Writing* CC (Table 3).

There appears to be no significant difference in PC2 between most polity-centuries regardless of supercluster membership (SI Fig. 2). Interestingly, however, the greatest variation in PC2 is seen in Cluster 2, the "transitionary" cluster. Whether this is perhaps a clue about the process that causes shifts in supercluster membership, or whether it bears any semantic meaning at all, is yet to be determined; further research and analysis is required.

Table 1. PCA eigenvalues

PC	Eigenvalue
1	7.5604
2	1.4152
3	0.7268
4	0.6183
5	0.4121
6	0.2549
7	0.0605
8	0.0227
9	0.0148

Table 2. Loadings for PC1

Rank #	CC	Loading
1	Hier	0.494
2	Money	0.459
3	PolTerr	0.400
4	PolPop	0.392
5	CapPop	0.326
6	Writing	0.297
7	Texts	0.137
8	Govt	0.095
9	Infra	0.089

Table 3. Loadings for PC2

Rank #	CC	Loading
1	Money	0.831
2	Writing	0.115
3	Texts	0.034
4	Infra	0.032
5	Govt	0.019
6	Hier	-0.231
7	PolTerr	-0.259
8	PolPop	-0.259
9	CapPop	-0.323

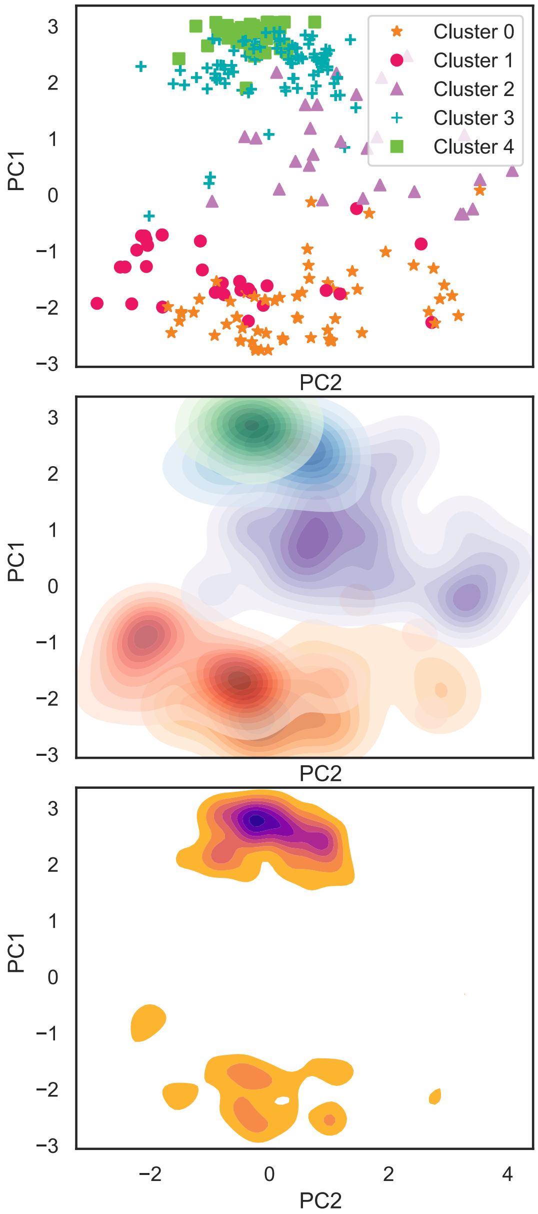

SI Fig. 2 PC2 plotted against PC1 with datapoints by cluster (top), per-cluster probability density (middle), and overall probability density (bottom).

References

1. Turchin P, Currie TE, Whitehouse H, François P, Feeney K, Mullins D, et al. Quantitative historical analysis uncovers a single dimension of complexity that structures global variation in human social organization. Proceedings of the National Academy of Sciences. 2018;115(2):E144–E151. doi:10.1073/pnas.1708800115.
