# Supplementary material for: The two types of society: Computationally revealing recurrent social formations and their evolutionary trajectories
Source: PLoS One. 2020 May 13;15(5):e0232609. doi: 10.1371/journal.pone.0232609 (PMC7219743; doi:10.1371/journal.pone.0232609)
Supplement: Attachment — Submitted filename: ResponseToReviewers.pdf [file pone.0232609.s007.pdf]

Joe Yuichiro Wakano, Ph.D.
Academic Editor
PLOS ONE

28 February 2020

Dear Dr. Wakano,

I am writing on behalf of myself and my coauthor, Dr. Jacob Freeman, along with the re-submission of our research article titled: "The two types of society: computationally revealing recurrent social formations and their evolutionary trajectories." Following the advice of the reviewers, we have clarified many of our methods, more rigorously addressed the goodness evaluation of our clustering, and included an exponential discount in equation 1, among other revisions. Please find a complete list of changes appended below in a point-by-point response to each reviewer.

I would also like to note that, since original submission, I have changed my first name and would like this to be reflected in the author list.

Thank you once again for considering our manuscript for publication. We believe that the paper makes a contribution to understanding the diversity of human societies and documenting trajectories of social-ecological evolution.

Sincerely and all the best,

Lux Miranda

Anthropology Program
Utah State University
jack.miranda@aggiemail.usu.edu

Specific Responses to Reviewers

Reviewer #1:

1. *In the manuscript, details for the method of DNN imputation is lacking. Which software did you use (e.g. chainer and tensorflow)? Which optimizer did you employ (e.g. momentum, adagrad, adadelta, etc.)? Moreover, DNN has many parameters: How many layers? How many units in each layer? How did you optimize hyperparameters? It would be nice if all the details are revealed so that one can easily replicate the present study.*

We agree that this was a much needed clarification. The section now explicitly identifies the imputation software, its structure, and our usage of it in a manner that should be suitable for replication. We have also clarified our language when referring to the software; since the DNN is the primary component but not the entire imputation tool itself (à la a car is not just its engine), we now instead refer to it as an "imputer."

2. *Lines 250-253: The authors wrote that PC1 computed by a PCA analysis using the same dataset was included in the input dataset for the main clustering analysis, and later the authors compared PC1 and the clustering result (Figs 2-6). I felt that it sounds tautological. Because the clustering uses information provided by PC1, PC1 and the clustering result is correlating as a matter of course. Removing PC1 from the input dataset should be scientifically better. Moreover, information contained in PC1 is contained in the other features (because PC1 is a summary of them) and therefore PC1 is redundant information. The SSC clustering should, in theory, reproduce the same result without PC1 information.*

We believe that sparse language on our part led to some confusion here. We do not include the PC1 metric as an input to clustering analysis; rather, the clustering is performed only on the 51-variable subset of Seshat's social complexity variables that has been used in prior analyses. That is, the PC1 column is removed from the dataset for the duration of clustering and is only added back afterwards to perform graphical analysis. One of the main points of our manuscript is to compare these distinct methods and identify if they allow us to see similar patterns. In short, the cluster analysis and PCA were performed separately on the same data, then we compared the results of the two methods. We have clarified this in the manuscript in the data and methods section.

3. *Line 252: The authors reported that PC1 contains only 61% of the total variance. Why not considering to look at higher PCs? For example, it may be interesting to draw Fig 2 in the PC1-PC2 plane and examine whether clusters 0 and 1 (3 and 4) are clearly separated.*

The manuscript now reports the variance explained by all higher PCs. We have also added an appendix that briefly explores the properties of PC2 and includes the suggested plot. Additionally, we realized that the 61% figure appears to have been a human error in transcribing the result from our program; the actual variance explained by PC1 is 68%. This is now corrected.

4. *Lines 309-310: "... find a clustering that minimizes the number of data points with a negative silhouette coefficient." I wonder if this criterion is statistically valid. There are a number of criteria to evaluate goodness of a non-supervised clustering result such as MDL, BIC, information entropy, etc. It could be more convincing to use one of them instead of the present one.*

We agree that this is perhaps an unsatisfying criterion to use, and the text now notes a recommendation for exploring other optimization criteria for future SSC optimization procedures.

5. *Lines 310-312: "... we finish by manually iterating over data points with a negative silhouette to relocate them to the cluster where they have the highest silhouette." It would be nice to describe this procedure in a computationally reproducible way. If the authors can manually optimize the clustering result, why the 'hyperopt' optimizer was not able to do so?*

We now include a more detailed description of this process suitable for algorithmic reproduction. We suggest that it is perhaps the choice of optimization criterion that did not lead to an optimal automatic clustering, thus necessitating the manual step.

6. *Around line 316: The authors may be able to evaluate the goodness of fit of the clustering by means of a*

bootstrapping method. Generating a number of shuffled dataset in each of which 271 cases are uniformly randomized for each feature, performing SSC clustering for each shuffled dataset, computing silhouette scores, and here we have a distribution of silhouette score if there were no significantly clustered structure. Using this, it would be possible to test whether or not the score 0.18 is significant.

We have implemented this method as described and report our results in the manuscript. We find that the mean silhouette score from clustering 540 random datasets is -0.15 and that the silhouette score exceeds 0.18 in 3.9% of cases.

Reviewer #2:

1. *In the title, "mathematically" seems to be the wrong word. Statistically? Computationally? A better word is needed.*

Agreed; we have chosen to instead use "computationally"

2. *"p-hacking" on p. 1: explain this term*

We have provided a brief explanation for those unfamiliar with the term.

3. *On p. 5, eqn (1). Shouldn't time be exponentially discounted, similarly to space? Yes, it would require an additional parameter. But remember that the depth of history that Seshat presents varies between different areas. If there is no discounting of distant past, long series (e.g., Anatolia) will accumulate large $x_{0,i,t}$ — and that doesn't make sense.*

This is a good point. We have included an exponential time discount for this term and re-ran the imputation process. Its effect on ρ_μ^2 is negligible, but we have updated the equation in the text.

4. *I don't understand how the "edge case" arises – how is it possible to have a bunch of identical Y_i? Does that result form multiple reuse of the same data point?*

We now include an explanation of how this edge case arises on lines 200-206. To summarize, it occurs during the k-fold cross-validation step of imputation when we happen to sample a subset of data where a certain column's entries all happen to be equal (typically an integer-valued column that has a small number of possible values, such as any of the hierarchy variables).

5. *I dislike "temperoculture" It evokes temperature, not time. Why not use the simpler polity-centuries?*

We agree that polity-centuries is a better term and have updated instances of the term accordingly.

6. *Fig. 1 seems to be missing silhouettes.*

The figure did have some cropping and color issues. These have been fixed.

7. *Fig. 7: time spent in each clusters is a useful diagnostic. But it would also be useful to see if superclusters 1 and 2 are attractors in the sense that most trajectories go "in" rather than "out".*

We have included a subplot showcasing this information in Fig. 7, having converted it to an a/b series.

8. *Finally, I dislike the juxtaposition of "Scale" vs. "Technology". The second is really misleading. Why not say simply "Non-Scale"?*

Agreed; we were using "Technology" to more closely mirror Peregrine's axes, but "Non-scale" is a much better descriptor. The text and plots have been updated accordingly.
