# Supplementary material for: The two types of society: Computationally revealing recurrent social formations and their evolutionary trajectories
Source: PLoS One. 2020 May 13;15(5):e0232609. doi: 10.1371/journal.pone.0232609 (PMC7219743; doi:10.1371/journal.pone.0232609)
Supplement: Attachment — Submitted filename: SecondResponseToReviewers.pdf [file pone.0232609.s008.pdf]

Joe Yuichiro Wakano, Ph.D.
Academic Editor
PLOS ONE

14 April 2020

Dear Dr. Wakano,

I am writing on behalf of myself and my coauthor, Dr. Jacob Freeman, along with the re-submission of our research article titled: "The two types of society: computationally revealing recurrent social formations and their evolutionary trajectories."

Following the advice of Reviewer 1, we have included the version number for the *datawig* package. We also fixed a minor redundancy of words in the first sentence of the abstract.

Thank you again for your work in reviewing this manuscript.

Sincerely and all the best,

Lux Miranda

Anthropology Program
Utah State University
jack.miranda@aggiemail.usu.edu
